# Chronological Age and Adaptive Outcomes Following Neuropsychomotor and Aquatic Interventions in Children with Autism: A Secondary Analysis

**DOI:** 10.3390/children13081081

**Published:** 2026-08-14

**Authors:** Martina Gnazzo, Giuditta Bargiacchi, Valentina Baldini, Maria Esposito, Rosa Passerini, Emanuela Varriale, Francesco Cerroni, Eva Germanò, Agata Maltese, Lucia Parisi, Michele Roccella, Giulia Spoto, Gabriella Di Rosa, Rita Barone, Lidia Scifo, Beatrice Gallai, Annamaria Maddalena Terracciano, Erminia Mannarino, Marco Carotenuto

**Affiliations:** 1 Clinic of Child and Adolescent Neuropsychiatry, Department of Mental Health, Physical and Preventive Medicine, University of Campania “Luigi Vanvitelli”, 80131 Naples, Italy; martina.gnazzo@unicampania.it (M.G.); giuditta.bargiacchi@studenti.unicampania.it (G.B.); maria.esposito3@unicampania.it (M.E.); francesco.cerroni@unicampania.it (F.C.); marco.carotenuto@unicampania.it (M.C.); 2 IRCCS Istituto delle Scienze Neurologiche di Bologna (ISNB), 40139 Bologna, Italy; 3 The PRINCE Network (Pediatric Regulatory and Integrative Neurodevelopmental Circuits and Endophenotypes), 80131 Naples, Italy; agata.maltese@unipa.it (A.M.); lucia.parisi@unipa.it (L.P.); michele.roccella@unipa.it (M.R.); gdirosa@unime.it (G.D.R.); rbarone@unict.it (R.B.); beatrice.gallai@unipg.it (B.G.); e.mannarino@aspcs.it (E.M.); 4 Department of Biomedical and Neuromotor Sciences, University of Bologna, 40126 Bologna, Italy; valentina.baldini7@unibo.it; 5 Centro di Riabilitazione LARS, 84087 Sarno, Italy; rosy@passerini.me (R.P.); e.varriale.tnpee@hotmail.it (E.V.); 6 Child and Adolescent Neuropsychiatry Unit, Department of Human and Evolutionary Pathology “Gaetano Barresi”, University of Messina, 98122 Messina, Italy; eva.germano@unime.it; 7 Department of Psychology, Educational Science and Human Movement, University of Palermo, 90128 Palermo, Italy; 8 Unit of Child Neurology and Psychiatry, Department of Biomedical Sciences, Dental Sciences and Morpho-Functional Imaging, University of Messina, 98125 Messina, Italy; 9 IRCCS Centro Neurolesi Bonino-Pulejo, 98124 Messina, Italy; 10 Child Neuropsychiatry Unit, Department of Clinical and Experimental Medicine, University of Catania, 95131 Catania, Italy; 11 Department of Law, Economics and Communication, LUMSA University, 00193 Roma, Italy; l.scifo@lumsa.it; 12 Department of Surgical and Biomedical Sciences, University of Perugia, 06123 Perugia, Italy; 13 Casa di Cura Santa Maria del Pozzo Hospital, 80049 Somma Vesuviana, Italy; amterracciano@gmail.com; 14 Child Neuropsychiatry, Azienda Sanitaria Provinciale (ASP), 87100 Cosenza, Italy

**Keywords:** autism spectrum disorder, neuroplasticity recruitment, developmental regulation, age moderation, neuropsychomotor therapy, TNPEE, aquatic therapy, adaptive functioning, Vineland Adaptive Behavior Scales, longitudinal study, pediatric rehabilitation

## Abstract

**Highlights:**

**What are the main findings?**

- TNPEE-based protocols yielded consistent adaptive improvements across the 4–12 year range, demonstrating therapeutic stability across distinct maturational stages.
- Adaptive functioning gains in the TAMA-only group followed a significant developmental gradient, showing limited effectiveness in children younger than six.

**What are the implications of the main findings?**

- These findings suggest that chronological age alone should not be considered a limiting factor for therapeutic responsiveness in children with ASD receiving structured developmental interventions.
- Treatment planning should prioritize intervention characteristics and individual developmental profiles rather than relying exclusively on chronological age.

**Abstract:**

**Background:** Autism Spectrum Disorder (ASD) can be conceptualized not as a static condition but as a dynamic disorder of developmental regulation, in which neuroplastic potential is translated into functional gains only through structured, experience-dependent therapeutic input. Neuropsychomotor Therapy of Early Development (TNPEE) and Therapy in Aquatic Motor Activities (TAMA) have shown differential efficacy in children with ASD, but whether chronological age moderates the magnitude of therapeutic response—or whether the characteristics of the intervention itself are the stronger determinant of outcome—has not been systematically examined. **Methods:** This was a non-randomized, exploratory secondary stratified analysis of a multicenter 18-month longitudinal cohort (N = 77 children with ASD, age 4–12 years) allocated to three groups: TAMA only, TNPEE combined with TAMA (TAMA+TNPEE), or TNPEE only. Participants were stratified into a younger (≤6 years, *n* = 16; TAMA n = 8, TAMA+TNPEE n = 3, TNPEE n = 5) and an older (>6 years, n = 61; TAMA n = 18, TAMA+TNPEE n = 22, TNPEE n = 21) subgroup. Adaptive functioning was assessed with the Vineland Adaptive Behavior Scales, Second Edition (VABS-II) at baseline and 18 months. Between-stratum comparisons used the Mann–Whitney U test; Spearman rank correlations and ANCOVA models with Age × Treatment interaction terms were used to test age moderation within a neuroplasticity-recruitment framework. **Results:** In the TAMA-only group, younger children showed significantly smaller adaptive gains than older children across the Composite (2.22 ± 1.14 vs. 3.53 ± 0.76, *p* = 0.012), Communication (*p* = 0.041), and Socialization (*p* = 0.016) subscales, with significant positive Spearman correlations between age and adaptive gains (r = 0.397–0.437). Simple slope analysis confirmed a significant age effect within the TAMA group (b = 0.231, SE = 0.083, *p* = 0.010) but not in the TNPEE (b = 0.133, *p* = 0.157) or TAMA+TNPEE (b = 0.135, *p* = 0.198) groups. The formal Age × Treatment interaction term in the ANCOVA did not reach statistical significance (all *p* > 0.18). As this interaction test is the formal statistical test of differential age moderation, the simple-slope pattern above should be regarded as exploratory rather than confirmatory. Treatment group accounted for the large majority of variance in adaptive gains (partial η^2^ = 0.78–0.95), whereas age explained only a small proportion of outcome variability. ASD severity level transitions were comparable across age strata in TNPEE-based groups. **Conclusions:** In this exploratory secondary analysis, TNPEE-based interventions were associated with adaptive gains that did not differ significantly by age across the 4–12 year age range, whereas aquatic therapy alone showed an age-dependent pattern favoring older children. These findings are hypothesis-generating and consistent with a neuroplasticity-recruitment model in which therapeutic architecture—rather than chronological age alone— may be associated with how effectively latent plastic potential is translated into developmental gains, although the non-significant Age × Treatment interaction and the small, imbalanced subgroups (including n = 3 in the youngest TAMA+TNPEE stratum) mean these findings require confirmation in adequately powered, prospectively designed studies, with implications for clinical decision-making regarding therapy timing and design.

## 1. Introduction

Autism Spectrum Disorder (ASD) is increasingly conceptualized not as a static neurodevelopmental condition, but as a dynamic disorder of developmental regulation, in which neural connectivity, sensory processing, cognitive learning, environmental experience, and social participation continuously interact across development [1,2]. Within this framework, developmental trajectories emerge from the reciprocal relationship between biological predispositions and experience-dependent mechanisms of adaptation [3], and motor, sensory, cognitive, emotional, and social functions should not be regarded as independent dimensions but as components of a dynamic, mutually interacting network. Contemporary models of thalamocortical function describe the brain as an active regulatory system in which sensory, cognitive, and emotional information is continuously selected, organized, and prioritized through dynamic cortical–subcortical interactions; from this perspective, developmental change can be understood as the outcome of an ongoing interaction between neuroplastic potential and the regulatory mechanisms that govern how experience is processed, integrated, and stabilized over time.

Neuroplasticity represents an intrinsic feature of the developing central nervous system and the core biological substrate enabling neurodevelopmental progression. It operates continuously through both spontaneous maturation and activity or experience-dependent interactions with the environment. However, although plastic capacity inherently unfolds without formal intervention, the presence of neuroplastic potential alone does not spontaneously yield clinically meaningful functional improvements. Structured therapeutic interventions serve to shape, organize, and consolidate adaptive neuroplastic processes by delivering targeted sensory, motor, cognitive, and social inputs, thereby promoting optimal developmental trajectories [4,5]. From this perspective, the effectiveness of an intervention may depend less on chronological age alone and more on its capacity to engage and organize neuroplastic processes—a structured neuroplasticity-recruitment strategy that provides the cognitive, sensory, motor, and social experiences necessary to transform latent plastic potential into measurable developmental change. The central question for therapy is therefore not simply whether neuroplasticity exists, or whether younger children possess greater neuroplastic potential, but how effectively that potential is recruited, organized, and stabilized through targeted developmental experiences.

A cornerstone of developmental neuroscience is the concept of synaptic plasticity—the capacity of neural circuits to modify their connectivity and functional organization in response to experience and learning [4,5]. Synaptic plasticity is most pronounced during early sensitive periods of brain development, typically concentrated in the first years of life, and progressively declines with age [6,7]. This neurobiological gradient underpins the widely held clinical assumption that early intervention maximizes therapeutic benefit in neurodevelopmental disorders, including ASD [8,9]. However, age may define the biological availability of neuroplastic potential without fully explaining treatment responsiveness: developmental outcomes appear to depend on how effectively therapeutic interventions recruit, channel, and stabilize neuroplastic mechanisms toward adaptive developmental trajectories, and the degree to which synaptic plasticity differences across the pediatric age range actually moderate therapeutic outcomes remains an underexplored empirical question.

Two intervention approaches have garnered increasing clinical and research attention in the therapy of children with ASD in Italy and Europe: Neuropsychomotor Therapy of Early Development (TNPEE, from the Italian Terapia della Neuro e Psicomotricità dell’Età Evolutiva) and Therapy in Aquatic Motor Activities (TAMA). TNPEE is a developmental-relational approach that targets the integration of motor, cognitive, emotional, and relational competencies through individualized, play-based, and sensorimotor activities, emphasizing the child as a unified psychophysical system, with interventions co-constructed within a relational context that involves both therapist and caregiver [10,11,12]. TAMA leverages the distinctive physical properties of water—buoyancy, hydrostatic pressure, and multisensory stimulation—to facilitate motor coordination, postural regulation, and sensory modulation [13,14,15,16,17,18].

A prior multicenter 18-month longitudinal study using the same cohort demonstrated that TNPEE, alone or combined with TAMA, produced significant improvements in core ASD symptomatology, adaptive functioning, sensory processing, and behavioral regulation, whereas TAMA alone was associated with improvements restricted to aquatic competence [19]. The sample included children ranging from 4 to 12 years of age—a window spanning substantially different phases of synaptic maturation—yet whether younger children benefited more from therapy than older counterparts, or whether the characteristics of the intervention itself were the stronger determinant of outcome, was not evaluated. Thus, while the parent study established the overall clinical efficacy of these interventions, it did not address whether the magnitude of therapeutic gains was contingent upon the child’s developmental stage or whether the architecture of the intervention could attenuate age-related differences in treatment response.

The present study therefore addresses this distinct and previously unexamined question through a secondary stratified analysis of the same multicenter dataset, examining whether treatment-related improvements in adaptive functioning are primarily associated with chronological age or with the characteristics of the therapeutic intervention itself. Using stratified group comparisons, Spearman correlations, and formal ANCOVA models with Age × Treatment interaction terms, we tested the hypothesis that neuroplasticity recruitment—the capacity of an intervention to activate, organize, and stabilize adaptive developmental processes—may represent a stronger determinant of developmental change than age alone. Specifically, we hypothesized that: (a) in TAMA-only, which relies more heavily on environmental enrichment, younger children in the high-plasticity window would show a smaller response, reflecting age-dependent recruitment; and (b) in TNPEE-based interventions, which directly target developmental-organizational mechanisms, therapeutic gains would be largely age-independent, reflecting a treatment architecture capable of engaging plasticity across a broader developmental window.

## 2. Materials and Methods

### 2.1. Ethical Statement

Ethical approval was granted by the Institutional Review Board of the University of Campania “Luigi Vanvitelli” (protocol no. 217, 4 August 2020). Written informed consent was obtained from the parents or legal guardians of all participants. The study was conducted in accordance with the Declaration of Helsinki.

### 2.2. Study Design and Parent Study

This is a secondary stratified analysis of data from a multicenter, 18-month longitudinal cohort study [19]. The parent study design, recruitment procedures, intervention protocols, and primary outcome analyses are reported in full elsewhere [19]. No additional data were collected; the current analysis focuses specifically on the moderating role of chronological age on adaptive functioning outcomes.

### 2.3. Participants

Seventy-seven children with ASD (30.6% female; age range 4–12 years, mean age 8.0 ± 2.1 years) were recruited from four clinical centers in Naples, Palermo, Perugia, and Sarno (Italy). Diagnosis was confirmed using the Autism Diagnostic Observation Schedule, Second Edition (ADOS-2) and the Childhood Autism Rating Scale, Second Edition (CARS-2), per DSM-5-TR criteria. All ADOS-2 assessments were conducted by experienced clinicians who had received formal training in the administration of the instrument and followed the standardized procedures described in the ADOS-2 manual. Inclusion required confirmed ASD diagnosis and age 2–12 years. Although the protocol allowed enrolment from 2 years of age, no children aged 2–3 years were recruited during the study period; the realized sample therefore spans 4–12 years. Exclusion criteria included significant sensory or motor impairment, chronic severe health conditions, and use of psychoactive medications.

For this stratified analysis, participants were classified into two age groups: ≤6 years (n = 16, a period of relatively high, though not sharply demarcated, synaptic density) and >6 years (n = 61), consistent with neurobiological evidence that the most pronounced phase of cortical synaptic density and experience-dependent plasticity occurs within the first six years of life [6,7]. Longitudinal neuroimaging studies have shown that the period from birth to approximately six years of age encompasses major phases of cortical growth, refinement, and reorganization, with relevant implications for subsequent cognitive and functional development [20]. In parallel, this age represents an important developmental transition, as fundamental motor, cognitive, and adaptive abilities progressively consolidate during early school age [21]. Therefore, stratification at six years allowed us to distinguish children within an early developmental phase characterized by rapid neural maturation from those in a subsequent phase of consolidation and refinement of neurodevelopmental skills. The age cutoff was established *a priori* based on the developmental literature reviewed above, rather than derived from the present dataset. Age was additionally modeled as a continuous variable in the ANCOVA analyses. The absence of sensitivity analyses using alternative age thresholds represents a limitation of the present study. Group allocation across age strata: TAMA only: ≤6 y n = 8, >6 y n = 18; TAMA+TNPEE: ≤6 y n = 3, >6 y n = 22; TNPEE only: ≤6 y n = 5, >6 y n = 21.

### 2.4. Interventions

Participants were allocated to one of three treatment conditions: (1) TNPEE only (three 50 min individual sessions per week); (2) combined TAMA+TNPEE (two neuropsychomotor plus one aquatic session weekly, each 50 min); or (3) TAMA only (three 50 min aquatic sessions per week). The three groups were matched for intervention intensity, with all participants receiving the same number of weekly sessions and the same session duration, differing only in the type of intervention provided. All interventions were conducted over 18 months by trained, certified professionals. Different therapists delivered the interventions across the participating centers; however, all were qualified TNPEE professionals with at least 15 years of clinical experience. Intervention goals and activities were tailored to each child’s developmental profile and therapeutic needs within a shared neuropsychomotor therapeutic framework. Although both TNPEE and TAMA concurrently engage motor, cognitive, socio-emotional, and communicative domains, they differ substantially in their therapeutic architecture and in the operational mechanisms driving developmental adaptation. TNPEE represents a structured, individualized neurodevelopmental approach in which the clinician actively scaffolds the child’s adaptive organization through dyadic co-regulation and progressively tailored neuropsychomotor experiences. Conversely, TAMA harnesses the unique biomechanical and multisensory properties of the aquatic environment—such as buoyancy, hydrostatic pressure, and thermal stimulation—to reduce gravitational constraints, promote sensorimotor regulation, and facilitate dynamic movement and functional participation in neurodevelopmental conditions. To enhance clarity for an international readership, the core operational similarities and distinctions between these two intervention modalities are synthesized in Table 1. Full intervention descriptions are reported in the parent study [19].

### 2.5. Outcome Measures

The primary outcome was adaptive functioning assessed by the Vineland Adaptive Behavior Scales, Second Edition (VABS-II) [22], a norm-referenced caregiver-interview instrument measuring Communication, Daily Living Skills, Socialization, Motor Skills, and a Composite score. VABS-II interviews were conducted and scored by experienced clinicians who were blinded to the treatment group allocation of the participants, following the assessment protocol established in the parent study. All VABS-II scores reported in this manuscript (Communication, Daily Living, Socialization, Motor, and Composite) are Standard Scores. Change scores (ΔT0 → T3 = T3 − T0) were computed for all subscales; positive values indicate improvement. Secondary outcomes included ASD severity level transitions (DSM-5-TR Levels 1–3) from baseline to 18 months.

The VABS-II was prospectively designated as the primary outcome measure for the moderation analyses because adaptive functioning represented the primary endpoint of the study. As a continuously distributed measure encompassing multiple developmental domains, the VABS-II is particularly well suited to evaluating treatment-by-age interactions. Accordingly, the present moderation analyses focused on VABS-II outcomes, whereas ADOS-2 was retained as a complementary secondary outcome to characterize changes in autism symptom severity.

### 2.6. Statistical Analysis

All analyses were conducted using SPSS 29.0 (IBM Corp., Armonk, NY, USA) and Python 3.11 (NumPy/SciPy). Descriptive statistics were computed for each subgroup defined by treatment condition and age stratum. Baseline comparability across six subgroups was assessed using one-way ANOVA for continuous variables and chi-square tests for categorical variables. The VABS-II Composite score was prespecified as the primary outcome. Analyses of the Communication, Daily Living, Socialization, and Motor subscales were considered secondary and exploratory and were therefore not adjusted for multiple comparisons. Regression coefficients are presented together with their 95% confidence intervals to convey the precision of the estimated effects; accordingly, subscale-level findings should be interpreted primarily in terms of effect estimates and their uncertainty rather than dichotomous statistical significance.

Within each treatment group, differences in VABS-II change scores between the ≤6 y and >6 y strata were evaluated using the Mann–Whitney U test (two-tailed). Spearman rank-order correlations between chronological age and VABS-II change scores were assessed within each treatment group.

To formally test whether the age–outcome association differed across treatment groups, we fitted ANCOVA models with Age × Treatment interaction terms. The model included: (1) chronological age, mean-centered at 8.02 years to reduce multicollinearity; (2) two treatment dummy variables (TNPEE and TAMA+TNPEE vs. TAMA as reference); and (3) two Age × Treatment product terms (Ageᴄ × TNPEE and Ageᴄ × TAMA+TNPEE). The joint significance of the interaction block was assessed via partial F-test comparing the full model to the main-effects-only model, with partial eta-squared (η^2^p) as effect size. Simple slopes—the regression of VABS-II gain on age within each treatment group separately—were extracted to characterize the direction and magnitude of age effects within each condition. A significance threshold of *p* < 0.05 was adopted throughout. Given the exploratory nature of the analysis and the known low power of interaction tests in small samples, all results are interpreted with appropriate caution.

To address baseline severity as a potential confounder, an additional ANCOVA for the VABS-II Composite change score was fitted including the mean-centered baseline VABS-II Composite score as a covariate alongside age, treatment, and the Age × Treatment interaction terms. To evaluate clustering by treatment center, the intraclass correlation coefficient (ICC) for the primary outcome was estimated across the four participating centers (Napoli, Perugia, Palermo, Sarno), and the main ANCOVA model was refitted with cluster-robust standard errors clustered by center; therapist identifiers were not retained in the anonymized dataset and could not be modeled. Ninety-five percent confidence intervals were additionally computed for the Mann–Whitney age-stratum comparisons (Hodges-Lehmann estimator with bootstrap CI, 10,000 resamples), the Spearman correlations (Fisher z-transformation), and the partial eta-squared effect sizes (non-central F distribution method). ASD severity-level transitions were additionally tested formally within each treatment group using a McNemar-Bowker test of marginal homogeneity on the T0 → T3 transition table and a Friedman test across all four repeated time points (T0–T3).

## 3. Results

### 3.1. Baseline Characteristics

Demographic and clinical characteristics by treatment group and age stratum are presented in Table 2. No statistically significant differences were observed across the six subgroups in sex distribution (*p* = 0.824), ADOS-2 (*p* = 0.783), CARS-2 (*p* = 0.612), or VABS-II Composite at baseline (*p* = 0.941). ASD severity level distribution was also comparable (*p* = 0.451), suggesting no evidence of baseline imbalance across strata; however, non-significance does not establish equivalence, and the small size of some subgroups (e.g., n = 3 in the TAMA+TNPEE ≤6 y stratum) limits the power of these comparisons to detect true differences.

### 3.2. VABS-II Change Scores by Age Stratum and Treatment Group

**Figure 1:**
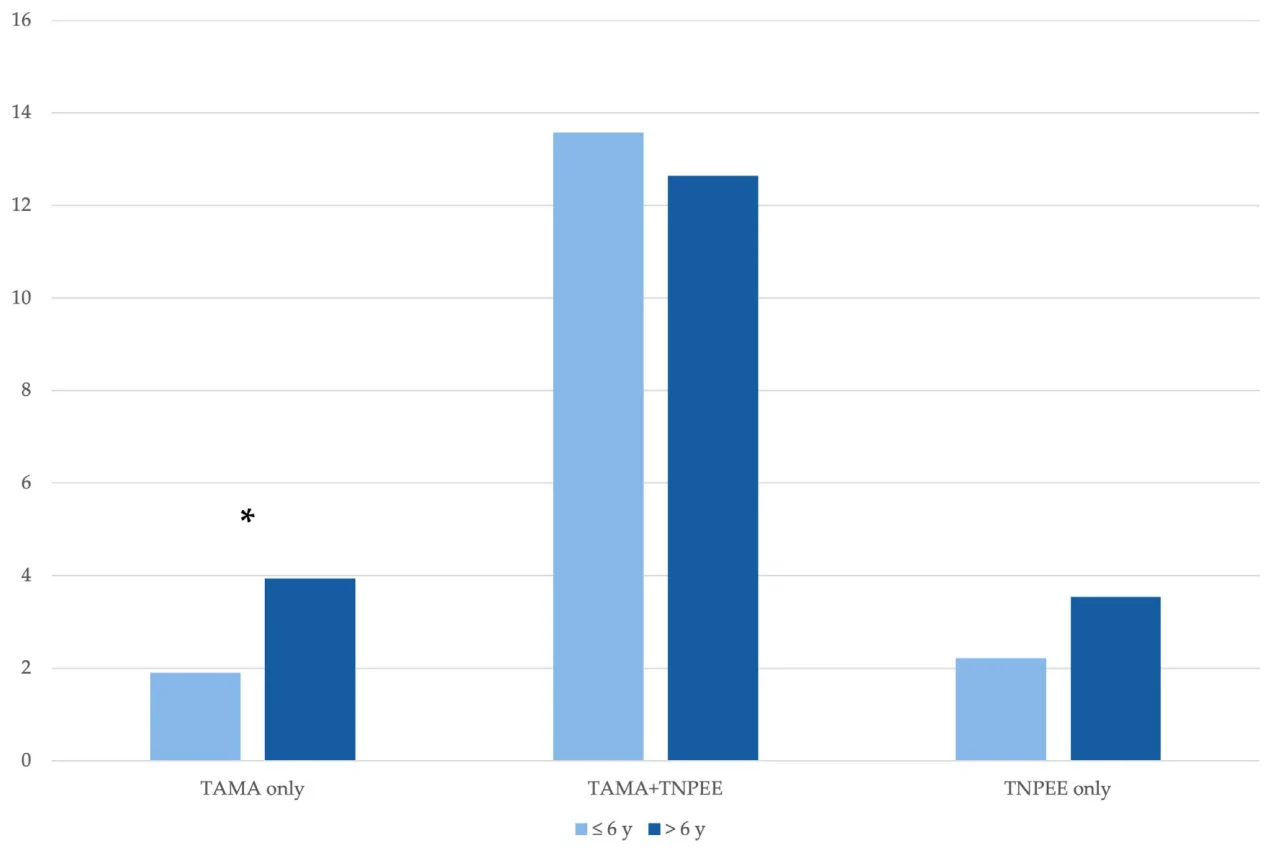
**Mean VABS-II Composite change scores (ΔT0 → T3) by treatment group and age stratum.** Bar heights = group means; error bars = ±1 SD. Blue = ≤6 years; dark blue = >6 years. Asterisk (*) = *p* < 0.05. Significant between-stratum difference in the TAMA group only (*p* = 0.012, Mann–Whitney U). No significant difference in TAMA+TNPEE (*p* = 0.969) or TNPEE (*p* = 0.705). Abbreviations: TAMA = Aquatic Motor Activities Therapy; TNPEE = Neuropsychomotor Therapy of Early Development; VABS-II = Vineland Adaptive Behavior Scales, Second Edition.

VABS-II change scores (ΔT0 → T3) stratified by treatment group and age stratum are reported in Table 3. In the TAMA-only group, statistically significant differences between age strata were observed for the Composite score (≤6 y: 2.22 ± 1.14 vs. >6 y: 3.53 ± 0.76, *p* = 0.012), Communication (1.90 ± 2.08 vs. 3.93 ± 1.64, *p* = 0.041), and Socialization (1.13 ± 1.71 vs. 3.18 ± 1.96, *p* = 0.016), with consistently smaller gains in younger children (Figure 1). No significant between-stratum differences were found for Daily Living or Motor in the TAMA group (both *p* > 0.70).

In the TAMA+TNPEE group, no significant age-stratum differences were detected across any VABS-II subscale (all *p* > 0.05), with mean Composite gains of 13.31 ± 1.17 (≤6 y) versus 13.20 ± 1.04 (>6 y) (*p* = 0.969). In the TNPEE-only group, no significant differences were observed between age strata for any subscale (all *p* > 0.25), with Composite gains of 10.23 ± 1.37 (≤6 y) versus 10.41 ± 0.86 (>6 y) (*p* = 0.705).

### 3.3. Spearman Correlations: Age and Adaptive Gains

**Figure 2:**
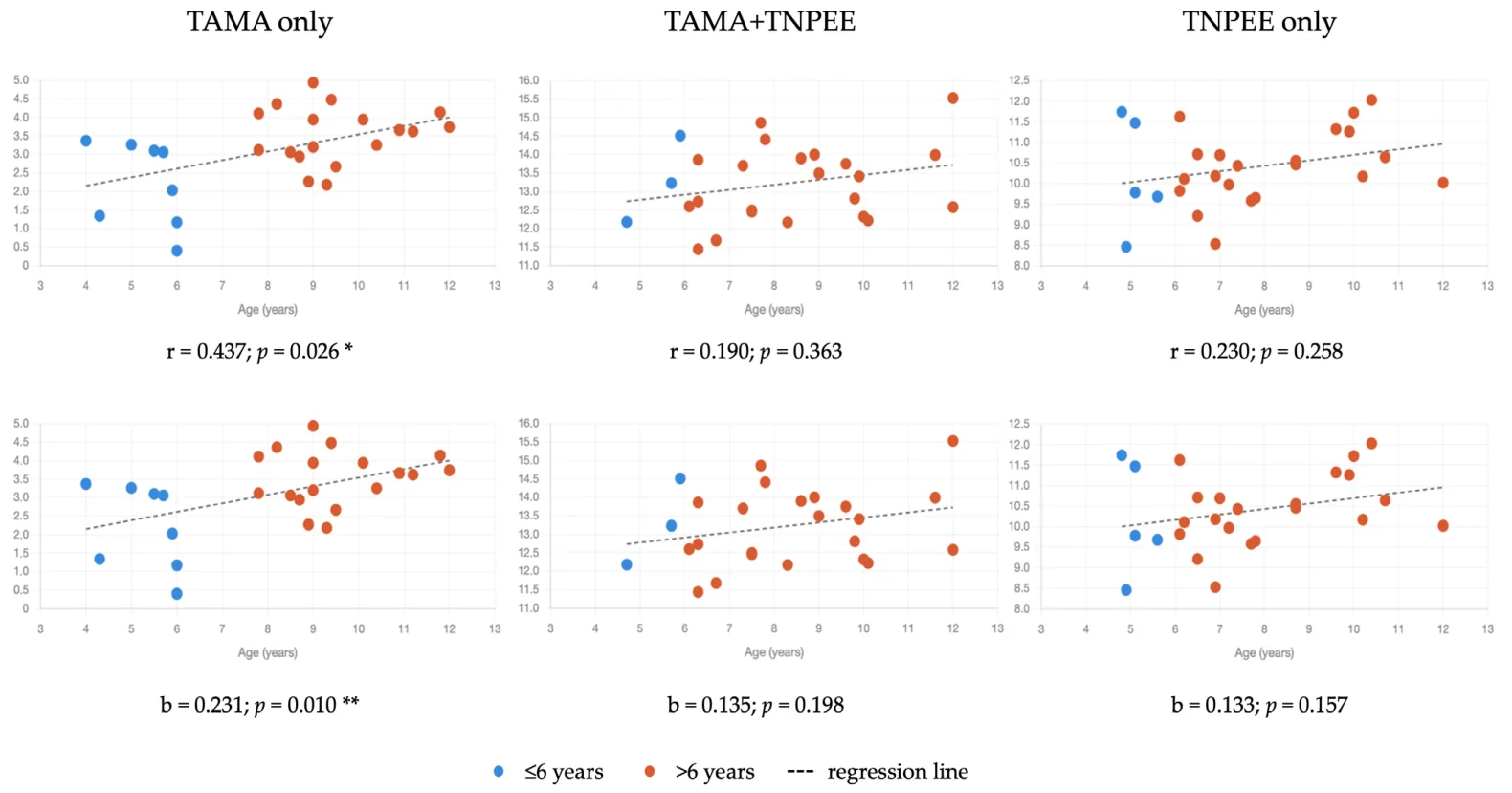
**Age–outcome relationship for the VABS-II Composite change score (ΔT0 → T3) by treatment group. Top** panel: Scatterplots of chronological age versus VABS-II Composite change score. Each point represents one participant. Blue indicates children ≤6 years and red indicates children >6 years. Dashed lines represent ordinary least-squares (OLS) regression fits. Spearman’s correlation coefficients (ρ) and corresponding *p* values are shown for each treatment group. Asterisk (*) = *p* < 0.05. A significant positive association was observed only in the TAMA group (ρ = 0.437, *p* = 0.026), whereas no significant associations were detected in the TAMA+TNPEE (ρ = 0.190, *p* = 0.363) or TNPEE-only (ρ = 0.230, *p* = 0.258) groups. **Bottom** panel: Simple linear regressions of VABS-II Composite change score on chronological age estimated separately within each treatment group. A significant positive age slope was observed only in the TAMA group (b = 0.231, SE = 0.083, *p* = 0.010), corresponding to an average increase of approximately 0.23 VABS-II Composite points per additional year of age. Asterisks (**) = *p* < 0.01. Regression slopes in the TAMA+TNPEE (b = 0.135, *p* = 0.198) and TNPEE-only (b = 0.133, *p* = 0.157) groups were smaller and not statistically significant. Abbreviations: TAMA = Aquatic Motor Activities Therapy; TNPEE = Neuropsychomotor Therapy of Early Development; VABS-II = Vineland Adaptive Behavior Scales, Second Edition.

**Figure 3:**
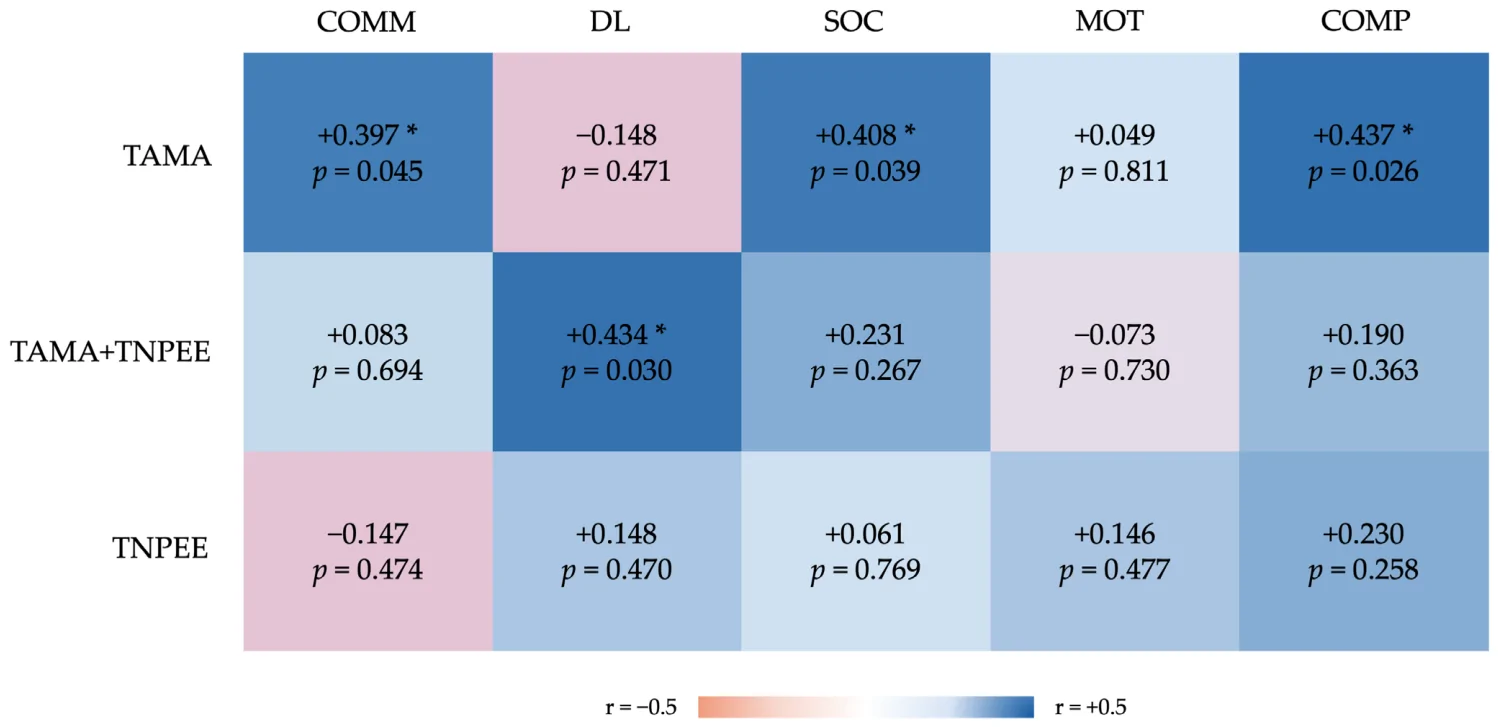
**Heatmap of Spearman rank correlations between chronological age and VABS-II subscale change scores across treatment groups.** Cell color encodes the Spearman rho coefficient: blue hues = positive correlations (older children gain more); orange hues = negative correlations (younger children gain more); white = near-zero. Asterisk (*) denotes *p* < 0.05. Significant positive correlations were observed predominantly in the TAMA-only group, for Communication (r = 0.397), Socialization (r = 0.408), and Composite (r = 0.437). No consistent age-related pattern was observed in the TNPEE groups. Abbreviations: COMM = Communication; COMP = Composite; DL = Daily Living; MOT = Motor; SOC = Socialization; TAMA = Aquatic Motor Activities Therapy; TNPEE = Neuropsychomotor Therapy of Early Development; VABS-II = Vineland Adaptive Behavior Scales, Second Edition.

Spearman rank correlations between chronological age and VABS-II change scores by treatment group are reported in Table 4 and illustrated in Figure 2 and Figure 3. In the TAMA-only group, significant positive correlations were observed for Communication (r = 0.397, *p* = 0.045), Socialization (r = 0.408, *p* = 0.039), and the Composite (r = 0.437, *p* = 0.026), indicating that older children showed greater adaptive gains under aquatic therapy alone. In the TAMA+TNPEE group, a significant correlation was observed for Daily Living (r = 0.434, *p* = 0.030) only. In the TNPEE-only group, no significant age-by-outcome correlation was detected for any subscale (all *p* > 0.25).

### 3.4. ANCOVA with Age × Treatment Interaction

Consistent with the analytic priority specified above, the Age × Treatment inter-action is treated here as the primary test of differential age moderation; the between-stratum comparisons and correlations reported in Section 3.2 and Section 3.3 are secondary, exploratory analyses. To formally test whether the slope of the age–outcome association differed across treatment groups, ANCOVA models including Age × Treatment interaction terms were fitted for each VABS-II subscale. Full model coefficients are reported in Table 5; results of the simple slope analysis (age effect estimated within each treatment group) are reported in Table 6; the main-effects ANCOVA with partial eta-squared is reported in Table 7.

The Age × Treatment interaction block did not reach statistical significance for any VABS-II subscale (all ΔF(2, 71) < 1.72, all *p* > 0.18, η^2^p < 0.05; Table 5). This result is consistent with limited statistical power to detect a moderately sized interaction in a sample of this size. The main-effects ANCOVA (Table 7) confirmed a significant main effect of treatment group for all subscales (all F(2, 73) > 131.00, all *p* < 0.001, η^2^p = 0.78–0.95), and a small but significant main effect of age for the Composite (F(1, 73) = 10.865, *p* = 0.002, η^2^p = 0.130), Communication (F(1, 73) = 4.760, *p* = 0.032, η^2^p = 0.061), and Socialization (F(1, 73) = 4.130, *p* = 0.046, η^2^p = 0.054) subscales, reflecting the positive age slope predominantly driven by the TAMA group.

Simple slope analysis (Table 6, Figure 2) revealed a significant positive regression of Composite gains on age within the TAMA group (b = 0.231, SE = 0.083, t = 2.80, *p* = 0.010), indicating that each additional year of age was associated with approximately 0.23 additional points of VABS-II Composite gain under aquatic therapy alone. The same association was also significant for Communication (b = 0.445, *p* = 0.007) and Socialization (b = 0.378, *p* = 0.035) within TAMA. In contrast, the age slope was non-significant in both the TNPEE group (Composite: b = 0.133, *p* = 0.157) and the TAMA+TNPEE group (Composite: b = 0.135, *p* = 0.198), with slopes numerically smaller than in the TAMA group. Taken together, the pattern of simple slopes supports an age-dependent gradient of adaptive gain specific to the aquatic-only condition, while TNPEE-based interventions show a flat, age-independent response profile. As the formal Age × Treatment interaction did not reach statistical significance, this simple-slope pattern should be regarded as exploratory and descriptive rather than as confirmatory evidence of differential age moderation.

### 3.5. Baseline-Adjusted and Clustering-Adjusted Sensitivity Analyses

To address whether baseline severity confounded the reported treatment and age effects, the primary ANCOVA model for the VABS-II Composite change score was refitted with the mean-centered baseline VABS-II Composite score included as an additional covariate. Baseline severity was not a significant predictor of the change score (B = −0.001, SE = 0.035, t = −0.04, *p* = 0.967, 95% CI [−0.069, 0.066]), and its inclusion left every other coefficient in the model essentially unchanged (Age: b = 0.232 vs. 0.231 in the unadjusted model; both Age × Treatment interaction terms remained non-significant, *p* > 0.44), consistent with the near-zero raw correlation between baseline VABS-II Composite and the change score (Pearson r = 0.019, *p* = 0.868). This indicates that the reported effects are not attributable to regression-to-the-mean or baseline-severity confounding. To address potential clustering by treatment center (four centers: Napoli, n = 35; Perugia, n = 16; Palermo, n = 16; Sarno, n = 10), the intraclass correlation coefficient (ICC) for the VABS-II Composite change score was estimated across centers and found to be effectively zero (ICC = −0.043; between-center F(3, 73) = 0.27, *p* = 0.848), indicating no meaningful center-level clustering. The main ANCOVA model was additionally refitted with cluster-robust standard errors clustered by center; treatment-group coefficients remained highly significant (all *p* ≤ 0.001) and the Age × Treatment interaction terms remained non-significant (both *p* > 0.54), closely matching the model-based standard errors. Given the small number of centers (k = 4), cluster-robust estimates should be interpreted with caution, as sandwich variance estimators are known to be imprecise with few clusters; nonetheless, the convergence of the ICC, cluster-robust, and model-based results provides consistent evidence against meaningful center-level confounding. Therapist identifiers were not retained in the anonymized dataset and could not be modeled.

### 3.6. ASD Severity Level Transitions

**Figure 4:**
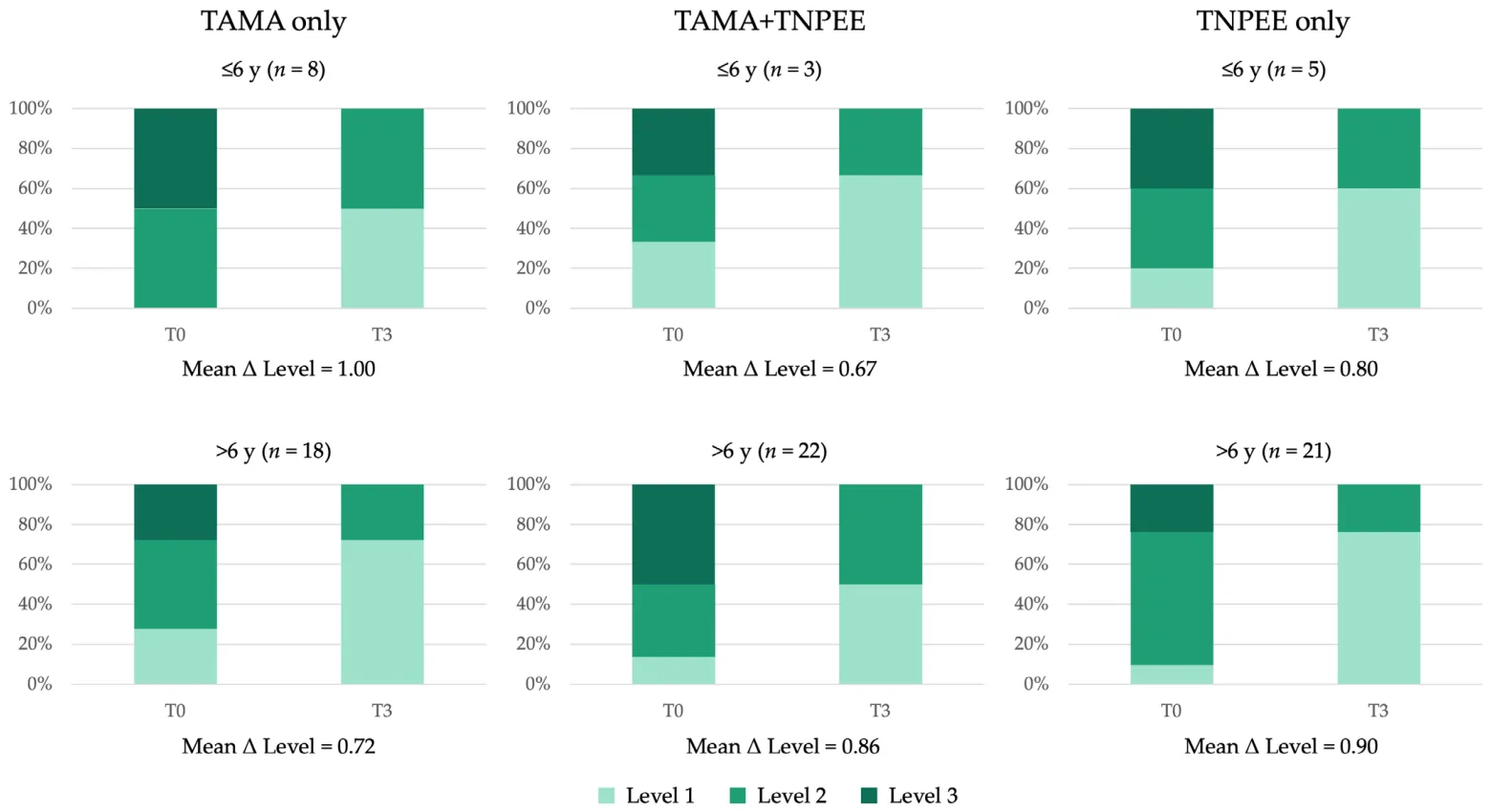
**ASD severity level distribution at T0 and T3, stratified by treatment group and age stratum.** Stacked bar charts showing the percentage of participants at each DSM-5-TR severity level (Level 1 = light teal; Level 2 = medium teal; Level 3 = dark teal). Six panels correspond to the six subgroups. In both age strata of the TNPEE-based groups, fewer participants were classified at Level 3 at T3 than at baseline. Mean Δ Level is reported per panel. Abbreviations: TAMA = Aquatic Motor Activities Therapy; TNPEE = Neuropsychomotor Therapy of Early Development.

ASD severity level transitions by treatment group and age stratum are reported in Table 8 and illustrated in Figure 4. In TNPEE-based groups, level transitions were comparable between age strata: TNPEE-only showed a mean Δ level of 0.80 ± 0.45 (≤6 y) versus 0.90 ± 0.30 (>6 y) (*p* = 0.557); TAMA+TNPEE showed 0.67 ± 0.58 (≤6 y) versus 0.86 ± 0.35 (>6 y) (*p* = 0.430). In both TNPEE groups, all Level 3 children at baseline transitioned to Level 2 or 1 by T3, regardless of age stratum. Using the severity-level data available at all four assessment time points (T0–T3), we additionally tested the overall T0 → T3 shift formally within each treatment group: a McNemar-Bowker test of marginal homogeneity on the 3 × 3 transition table, and a Friedman test across all four repeated time points. Both tests were highly significant in every group (McNemar-Bowker: χ^2^ = 21.0–23.0, df = 2, all *p* < 0.0001; Friedman: χ^2^ = 36.9–42.3, df = 3, all *p* < 0.0001; full statistics in Table 7), confirming that the reduction in severity level over time was unlikely to be due to chance in every treatment arm. Because DSM-5-TR severity levels are based on clinical judgement rather than an interval-scaled continuous measure, and because documentation regarding rater blinding was unavailable in this secondary analysis, observed severity-level transitions should be considered descriptive and exploratory findings.

In the TAMA-only group, a descriptive trend was observed (mean Δ level: ≤6 y = 1.00 ± 0.00 vs. >6 y = 0.72 ± 0.46, *p* = 0.113), not reaching statistical significance, and attributable in part to the absence of Level 1 children in the ≤6 y TAMA subgroup at baseline.

## 4. Discussion

This secondary stratified analysis of an 18-month multicenter longitudinal dataset examined whether chronological age moderated adaptive functioning gains among children with ASD receiving TNPEE, TAMA+TNPEE, or TAMA alone, and whether the pattern of results was better explained by a neuroplasticity-recruitment framework than by age alone. Three complementary analytical approaches—stratified Mann–Whitney comparisons, Spearman correlations, and ANCOVA with Age × Treatment interaction terms—converged on a consistent pattern: TNPEE-based interventions were associated with adaptive gains that did not differ significantly by age across the 4–12 year range, whereas TAMA alone showed an age-dependent profile with significantly smaller adaptive gains in younger children. As detailed below, the formal Age × Treatment interaction test was non-significant, and the findings should accordingly be read as hypothesis-generating.

The formal ANCOVA interaction test did not reach statistical significance (all *p* > 0.18), a result that must be interpreted in light of well-established limitations of interaction testing in observational studies with modest sample sizes [23,24]. The statistical power to detect a significant Age × Treatment interaction is substantially lower than the power to detect main effects, typically requiring sample sizes two to four times larger [23]. The small but consistent simple-slopes pattern—a significant age effect in TAMA (b = 0.231, *p* = 0.010) and flat, non-significant slopes in both TNPEE groups (b ≈ 0.133–0.135, *p* > 0.15)—constitutes exploratory, descriptive evidence—rather than confirmatory evidence—of differential age moderation, and should be interpreted with caution given the non-significant formal interaction term. Effect sizes for the interaction block (η^2^p = 0.010–0.046) are consistent with a small-to-moderate moderation effect that would require approximately 150–200 participants to detect with adequate power.

Critically, the main-effects ANCOVA indicated that treatment group accounted for the vast majority of variance in adaptive gains (partial η^2^ ranging from 0.78 to 0.95), whereas chronological age explained only a small proportion of outcome variability, reaching significance only for the Composite, Communication, and Socialization subscales (η^2^p = 0.054–0.130), and driven predominantly by the TAMA group. No significant Age × Treatment interaction was observed for any Vineland domain. Taken together, these findings indicate that treatment characteristics were substantially more influential than developmental age in determining clinical outcome. Rather than supporting a purely age-dependent model of improvement, the present data are more consistent with a neuroplasticity-recruitment framework, in which therapeutic architecture, developmental organization, and environmental enrichment play a greater role than chronological age alone in shaping adaptive developmental trajectories. It should be emphasized that this neuroplasticity-recruitment interpretation remains a conceptual hypothesis inferred from the observed behavioral pattern, rather than a directly demonstrated biological mechanism; substantiating it would require dedicated neuroimaging or electrophysiological investigation.

The age-dependent pattern observed in the TAMA group is counterintuitive from a classical plasticity perspective, in which younger children—presumed to have greater plastic potential—would be expected to benefit most. One plausible interpretation, consistent with a neuroplasticity-recruitment model, is that TAMA primarily provides multisensory stimulation, motivational enhancement, emotional engagement, and opportunities for embodied social participation—an environmental enrichment strategy that increases the availability of plasticity-related processes without itself organizing them. Engaging with this enriched environment may rely to a greater degree on the child’s existing cognitive and executive capacities—including attentional regulation, motor learning, and social anticipation—which develop progressively with age [23,24,25]. Younger children with ASD may therefore derive fewer adaptive benefits from aquatic therapy alone not because of lower plasticity, but because the intervention’s efficacy is partially contingent on capacities not yet fully available under age 6, consistent with prior aquatic therapy literature suggesting that clinical generalization from aquatic environments requires cognitive scaffolding not intrinsic to water-based motor activities [13,17].

In contrast, the absence of a statistically significant age association within the TNPEE and TAMA+TNPEE simple slopes is a pattern that merits further theoretical consideration. TNPEE is grounded in a developmental-relational framework emphasizing individualized co-regulation, embodied interaction, and the progressive construction of adaptive competencies within a responsive therapeutic relationship [10,11,12]. Unlike TAMA, TNPEE directly targets developmental functions central to the organization of adaptive behavior—motor planning, symbolic functioning, executive regulation, attentional control, social reciprocity, adaptive problem solving, and relational competence—and adapts to the child’s current functional level rather than requiring a threshold of cognitive or executive maturity. Within a neuroplasticity-recruitment framework, TNPEE may plausibly contribute not simply by enhancing neuroplasticity, but by facilitating its organization toward functionally meaningful developmental outcomes across a broad maturation continuum, irrespective of whether the child is 5 or 10 years old. This study measured caregiver-reported adaptive behavior only and did not directly assess synaptic plasticity, neural recruitment, sensitive-period regulation, excitation–inhibition balance, neural network maturation, or any other neurophysiological index; the neuroplasticity-recruitment interpretation therefore remains speculative and should be evaluated alongside the alternative, non-mutually exclusive explanations discussed below.

This interpretation may help explain why the combined TAMA+TNPEE intervention yielded the largest overall improvements in this cohort. The superior outcomes observed in the TAMA+TNPEE group may reflect the convergence of two complementary mechanisms: environmental enrichment provided by aquatic therapy and developmental organization provided by neuropsychomotor intervention. In this model, TAMA increases the richness and salience of developmental experiences, whereas TNPEE channels these experiences toward the acquisition of adaptive, cognitive, social, and behavioral competencies. The therapeutic benefit therefore appears to arise not simply from increased treatment intensity, but from the interaction between plasticity enhancement and plasticity organization.

Several alternative, non-mutually exclusive explanations for the observed pattern should be considered and could not be disentangled within the present dataset, including differences in treatment content between TNPEE and TAMA; non-random treatment allocation or referral pathways; variations in therapist–child interaction and therapeutic alliance; caregiver engagement; expectancy or rater effects; normative developmental maturation over the 18-month follow-up; center-specific influences; and unmeasured concomitant interventions. Discriminating among these explanations, and between them and the neuroplasticity-recruitment hypothesis, will require adequately powered prospective studies incorporating randomization, rigorous treatment-fidelity monitoring, detailed characterization of intervention exposure, and objective neurophysiological biomarkers of treatment response.

These findings complement the growing literature on early intervention in ASD [8,9] and evidence on the efficacy of structured neuropsychomotor interventions [26,27,28,29,30,31], while challenging a strict interpretation of the early-intervention imperative. They suggest that structured neuropsychomotor interventions can support clinically meaningful adaptive gains across a broader developmental period than traditionally assumed. While early intervention remains the clinical standard, our results indicate that chronological age alone does not fully determine therapeutic responsiveness. Early diagnosis remains essential because it provides access to sensitive developmental windows; however, the effectiveness of TNPEE-based intervention is not reducible to age alone, and the quality and architecture of the intervention may ultimately determine how effectively that opportunity is transformed into long-term developmental gains.

The convergent evidence from ASD severity level transitions further reinforces the adaptive functioning findings: in TNPEE-based groups, the proportion of children transitioning from higher to lower severity levels was statistically comparable between age strata. Several studies have reported atypical synaptic development trajectories in ASD, including persistent alterations in excitatory/inhibitory balance [4,5,7,32,33,34,35], which may extend the window of experience-dependent plasticity beyond typical development. Within the framework of ASD as a disorder of sensitive-period regulation—in which the timing, intensity, duration, opening, or closure of sensitive developmental windows may be altered rather than plasticity being simply diminished with age [6]—the neuroplasticity window may not close as abruptly as classical models predict, potentially explaining the sustained TNPEE efficacy across the age range studied here. The following biological parallel is offered as a speculative avenue for future mechanistic research rather than as an explanation supported by the present behavioral data: recent multi-omics studies have further reinforced the concept that ASD, despite its remarkable genetic and clinical heterogeneity, converges on a limited number of shared biological pathways, including synaptic plasticity, excitation-inhibition balance, neural network maturation, neuroinflammation, and mitochondrial regulation [36,37,38]; developmental interventions such as TNPEE and TAMA may therefore be conceptualized as convergence-oriented strategies capable of influencing adaptive outcomes despite this substantial biological heterogeneity.

### Limitations and Future Directions

Several limitations warrant consideration. First, the formal Age × Treatment interaction did not reach significance, limiting the strength of causal inference about age moderation; all findings should be considered exploratory. Second, although the three treatment groups were well balanced in overall size (TAMA, n = 26; TAMA+TNPEE, n = 25; TNPEE, n = 26), stratification by age resulted in relatively small younger-age subgroups (n = 3–8 within treatment arms), substantially reducing power for moderation and interaction analyses. In particular, the TAMA+TNPEE ≤6 y subgroup (n = 3) is too small to provide stable estimates, and findings for the ≤6 y stratum as a whole should be considered preliminary pending replication in adequately powered, prospectively stratified cohorts. Third, the six-year cutoff represents an approximate developmental threshold. Although informed by developmental and neurobiological considerations, neuroplasticity does not operate as a discrete process beginning or ending at a specific age. Alternative cutoffs and nonlinear continuous-age models were not examined, and the available documentation does not allow independent verification of the timing of the age-moderation analysis plan. Fourth, the non-randomized design of the parent study introduces potential confounding related to clinical characteristics, treatment setting, therapist-related factors, family characteristics, concomitant services, and other unmeasured variables. Accordingly, the large treatment-associated effect sizes observed in the present analysis (partial η^2^ = 0.78–0.95) should be interpreted cautiously. Natural developmental maturation over the 18-month follow-up may also have contributed to the observed gains, particularly among younger children, given the absence of an untreated or active control condition.

Further limitations arise from the retrospective secondary nature of the present analysis and the availability of only an anonymized summary dataset. The dataset did not contain therapist identifiers or sufficiently granular information on treatment fidelity, attendance, session-level intervention characteristics, adverse events, or concomitant services. Therapist-level clustering could therefore not be modeled, and the contribution of these factors to treatment effects could not be evaluated retrospectively. Although ADOS-2 assessments were performed by formally trained clinicians following standardized administration procedures, the documentation retained for this secondary analysis did not include information regarding assessor blinding, assessor continuity across time points, or formal inter-rater reliability procedures. These methodological details could therefore not be verified retrospectively and should be considered when interpreting the findings. The multiplicity of inferential analyses also increases the possibility of chance findings, although all analyses were explicitly considered exploratory. No prospective or sensitivity power analysis based on the observed interaction effect size was performed; such analyses should be incorporated into future studies.

Finally, the neuroplasticity-recruitment interpretation is conceptual rather than directly biological. The study assessed clinical and caregiver-reported adaptive outcomes but did not measure synaptic plasticity, neural recruitment, network maturation, or other neurobiological indices. The proposed framework should therefore be regarded as one possible interpretation of the observed clinical pattern, alongside alternative explanations related to treatment selection, intervention content, therapist attention, caregiver involvement, measurement expectancy, maturation, center effects, and unreported concurrent services. Future studies should combine adequately powered, prospectively defined age strata with randomized or active-comparator designs and longitudinal neuroimaging or electrophysiological measures to directly test whether neuroplasticity-related mechanisms contribute to differential therapeutic response.

## 5. Conclusions

In this exploratory secondary analysis, age was positively associated with selected adaptive gains in the TAMA-only group, whereas no statistically significant age associations were detected in the TNPEE-based groups. However, formal Age × Treatment interactions were non-significant, and the small, imbalanced subgroups preclude firm conclusions regarding differential age moderation. These patterns warrant prospective evaluation in adequately powered randomized studies. More broadly, these findings are compatible with—but do not confirm—a developmental model in which ASD is characterized not simply by altered plasticity, but by dysregulation of the mechanisms governing sensitive developmental windows. The relevant clinical question for future, adequately powered research is therefore not merely whether younger children improve more, but whether a given intervention can recruit, organize, and sustain adaptive neuroplasticity beyond the influence of chronological age alone.

## Figures and Tables

**Table 1:** Comparative characteristics of TNPEE and TAMA.

Dimension	TNPEE (Neuropsychomotor Therapy of Early Development)	TAMA (Therapy in Aquatic Motor Activities)
Primary therapeutic philosophy	Structured, developmental-relational intervention promoting multi-domain adaptive organization	Environmental enrichment intervention leveraging the aquatic medium as a multisensory therapeutic context
Theoretical and Biological Framework	Neurodevelopmental regulation through dyadic interaction and tailored neuropsychomotor experiences	Sensorimotor facilitation driven by the biomechanical and physical properties of water (e.g., buoyancy, hydrostatic pressure)
Therapeutic Setting	Individualized clinical neuropsychomotor room	Purpose-designed therapeutic swimming pool
Core Target Domains	Integrated motor, cognitive, socio-emotional, and communicative-linguistic functions	Motor coordination, postural control, sensorimotor integration, emotional regulation, and body awareness
Therapeutic Strategy	Structured, individualized, goal-directed activities progressively adapted to the child’s developmental profile	Guided engagement within an enriched aquatic environment that facilitates movement execution and active participation
Role of the therapist	Active scaffolding via dyadic co-regulation and progressive adaptation of task complexity	Facilitation of motor exploration, environmental adaptation, and social interaction within the water
Family involvement	Core systemic component aimed at supporting skill generalization in home/daily environments	Directed toward enhancing engagement, treatment adherence, and functional skill transfer to everyday life
Mechanism of Action	Direct recruitment and structuring of neuroplastic processes through clinician-guided, experience-dependent learning	Indirect modulation of neuroplasticity via multisensory stimulation, postural unloading, and heightened motivation

Both interventions engage motor, cognitive, emotional, and social processes. Their primary distinction lies not in the functional domains they address, but in the therapeutic architecture through which developmental adaptation is promoted. TNPEE primarily relies on individualized developmental organization and relational scaffolding, whereas TAMA capitalizes on the multisensory and physical properties of the aquatic environment to facilitate adaptive change.

**Table 2:** Baseline demographic and clinical characteristics by treatment group and age stratum.

Variable	TAMA ≤ 6 y (n = 8)	TAMA > 6 y (n = 18)	TAMA+TNPEE ≤ 6 y (n = 3)	TAMA+TNPEE > 6 y (n = 22)	TNPEE ≤ 6 y (n = 5)	TNPEE > 6 y (n = 21)	*p*-Value
Age, mean ± SD (y)	5.3 ± 0.8	9.5 ± 1.3	5.4 ± 0.6	8.6 ± 1.9	5.1 ± 0.3	8.2 ± 1.8	—
Female, n (%)	3 (38%)	4 (22%)	1 (33%)	8 (36%)	1 (20%)	7 (33%)	0.824
ADOS-2 Total	19 ± 2.2	16.9 ± 2.4	17 ± 0.1	17.1 ± 2.2	17.9 ± 2.7	17.5 ± 1.6	0.296
VABS-II Composite	52.3 ± 4.0	52.8 ± 3.6	52.3 ± 5.6	53.0 ± 2.2	51.6 ± 1.4	52.8 ± 3.7	0.941
ASD Level 1, n (%)	0 (0%)	5 (28%)	1 (33%)	3 (14%)	1 (20%)	2 (10%)	0.451
ASD Level 2, n (%)	4 (50%)	8 (44%)	1 (33%)	8 (36%)	2 (40%)	14 (67%)	
ASD Level 3, n (%)	4 (50%)	5 (28%)	1 (33%)	11 (50%)	2 (40%)	5 (24%)	

Data are mean ± SD or n (%). Between-group comparisons: one-way ANOVA for continuous variables, chi-square for categorical. ASD Level classification per DSM-5-TR criteria (Level 1 = requires support; Level 2 = requires substantial support; Level 3 = requires very substantial support). Abbreviations: ADOS-2 = Autism Diagnostic Observation Schedule, 2nd ed.; ASD = Autism Spectrum Disorder; CARS-2 = Childhood Autism Rating Scale, 2nd ed.; TAMA = Aquatic Motor Activities Therapy; TNPEE = Neuropsychomotor Therapy of Early Development; VABS-II = Vineland Adaptive Behavior Scales, Second Edition.

**Table 3:** VABS-II subscale change scores (ΔT0 → T3) by treatment group and age stratum.

VABS-II Subscale	TAMA ≤ 6 y (n = 8)	TAMA > 6 y (n = 18)	*p*-Value	TAMA+TNPEE ≤ 6 y (n = 3)	TAMA+TNPEE > 6 y (n = 22)	*p*-Value
VABS-II Communication	1.90 ± 2.08	3.93 ± 1.64	0.041 * HL −1.52 [−3.79, −0.11]	13.58 ± 1.24	12.64 ± 1.83	0.398 HL 0.79 [−0.19, 2.49]
VABS-II Daily Living	3.24 ± 2.55	3.62 ± 1.76	0.724 HL −0.58 [−2.33, 1.92]	14.55 ± 4.49	13.86 ± 1.72	0.900 HL −0.40 [−3.11, 5.33]
VABS-II Socialization	1.13 ± 1.71	3.18 ± 1.96	0.016 * HL −1.85 [−3.54, −0.42]	11.65 ± 0.36	13.72 ± 2.41	0.058 HL 0.31 [−0.86, 2.27]
VABS-II Motor	2.59 ± 2.28	3.41 ± 2.37	0.765 HL −0.30 [−2.62, 1.36]	13.45 ± 0.43	12.58 ± 2.48	0.906 HL 0.31 [−0.86, 2.27]
VABS-II Composite	2.22 ± 1.14	3.53 ± 0.76	0.012 * HL −1.10 [−2.34, −0.41]	13.31 ± 1.17	13.20 ± 1.04	0.969 HL 0.05 [−0.97, 1.29]
	TNPEE ≤ 6 y (n = 5)	TNPEE > 6 y (n = 21)	*p*-value			
VABS-II Communication	10.60 ± 0.82	10.29 ± 2.10	0.659 HL 0.18 [−1.04, 1.64]			
VABS-II Daily Living	9.15 ± 2.43	10.51 ± 1.87	0.447 HL −0.84 [−3.84, 0.85]			
VABS-II Socialization	11.52 ± 1.38	10.62 ± 2.11	0.250 HL 0.98 [−1.00, 1.90]			
VABS-II Motor	9.63 ± 2.92	10.23 ± 1.57	0.705 HL −0.59 [−3.30, 1.90]			
VABS-II Composite	10.23 ± 1.37	10.41 ± 0.86	0.705 HL −0.26 [−1.56, 1.20]			

Data are mean ± SD. Positive values indicate improvement. Between-age-stratum comparisons: Mann–Whitney U test (two-tailed). Asterisk (*) denotes *p* < 0.05. Hodges-Lehmann estimate of the location shift (≤6 y minus >6 y), with 95% bootstrap confidence interval (10,000 resamples), reported alongside each *p*-value. Abbreviations: TAMA = Aquatic Motor Activities Therapy; HL = Hodges-Lehmann estimate; TNPEE = Neuropsychomotor Therapy of Early Development; VABS-II = Vineland Adaptive Behavior Scales, Second Edition.

**Table 4:** Spearman rank correlations between chronological age and VABS-II subscale change scores (ΔT0 → T3) by treatment group.

**VABS-II Subscale**	**TAMA (n = 26)**			**TAMA+TNPEE (n = 25)**			**TNPEE (n = 26)**		
	**r**	***p*** **-Value**	**Sig.**	**r**	***p*** **-Value**	**Sig.**	**r**	***p*** **-Value**	**Sig.**
VABS-II Communication	0.397 [0.01, 0.68]	0.045	*	0.083 [−0.32, 0.46]	0.694	—	−0.147 [−0.51, 0.26]	0.474	—
VABS-II Daily Living	−0.148 [−0.51, 0.25]	0.471	—	0.434 [0.05, 0.71]	0.030	*	0.148 [−0.25, 0.51]	0.470	—
VABS-II Socialization	0.408 [0.02, 0.69]	0.039	*	0.231 [−0.18, 0.57]	0.267	—	0.061 [−0.34, 0.44]	0.769	—
VABS-II Motor	0.049 [−0.35, 0.43]	0.811	—	−0.073 [−0.46, 0.33]	0.730	—	0.146 [−0.26, 0.51]	0.477	—
VABS-II Composite	0.437 [0.06, 0.71]	0.026	*	0.190 [−0.22, 0.54]	0.363	—	0.230 [−0.17, 0.57]	0.258	—

Spearman’s rho (r) and *p*-values reported. 95% confidence intervals (Fisher z-transformation) are reported in brackets alongside each r. Asterisk (*) denotes *p* < 0.05. Positive r = older children gain more; negative r = younger children gain more. Non-significant correlations marked with em-dash (—). Abbreviations: TAMA = Aquatic Motor Activities Therapy; TNPEE = Neuropsychomotor Therapy of Early Development; VABS-II = Vineland Adaptive Behavior Scales, Second Edition.

**Table 5:** ANCOVA with Age × Treatment interaction: full model coefficients for VABS-II change scores (ΔT0 → T3).

Predictor	VABS-II Composite				VABS-II Communication				VABS-II Socialization			
	B	SE	t	*p*	B	SE	t	*p*	B	SE	t	*p*
Intercept	3.081 [2.709, 3.453]	0.190	16.25	<0.001 ***	3.215 [2.509, 3.921]	0.360	8.92	<0.001 ***	2.473 [1.654, 3.292]	0.418	5.92	<0.001 ***
Age (centered)	0.231 [0.066, 0.396]	0.084	2.75	0.008 **	0.445 [0.131, 0.759]	0.160	2.79	0.007 **	0.378 [0.015, 0.741]	0.185	2.04	0.045 *
TNPEE vs. TAMA	7.350 [6.821, 7.879]	0.270	27.19	<0.001 ***	7.141 [6.134, 8.148]	0.514	13.90	<0.001 ***	8.365 [7.199, 9.531]	0.595	14.05	<0.001 ***
TAMA+TNPEE vs. TAMA	10.104 [9.573, 10.635]	0.271	37.29	<0.001 ***	9.512 [8.503, 10.521]	0.515	18.48	<0.001 ***	10.961 [9.791, 12.131]	0.597	18.37	<0.001 ***
Age × TNPEE	−0.099 [−0.348, 0.150]	0.127	−0.78	0.439	−0.424 [−0.896, 0.048]	0.241	−1.76	0.083	−0.265 [−0.812, 0.282]	0.279	−0.95	0.346
Age × TAMA+TNPEE	−0.096 [−0.347, 0.155]	0.128	−0.75	0.456	−0.315 [−0.791, 0.161]	0.243	−1.29	0.200	−0.209 [−0.762, 0.344]	0.282	−0.74	0.462
Model fit	R^2^ = 0.955		F(5,71) = 301.67		R^2^ = 0.842		F(5,71) = 75.61		R^2^ = 0.841		F(5,71) = 75.01	
Interaction block	ΔF(2,71) = 0.407		*p* = 0.667, η^2^p = 0.011		ΔF(2,71) = 1.722		*p* = 0.186, η^2^p = 0.046		ΔF(2,71) = 0.516		*p* = 0.599, η^2^p = 0.014	
**Predictor**	**VABS-II Daily Living**						**VABS-II Motor**					
	**B**		**SE**	**t**	***p***		**B**		**SE**	**t**	***p***	
Intercept	3.510 [2.730, 4.290]		0.398	8.83	<0.001 ***		3.124 [2.273, 3.975]		0.434	7.19	<0.001 ***	
Age (centered)	−0.049 [−0.394, 0.296]		0.176	−0.28	0.782		0.152 [−0.226, 0.530]		0.193	0.79	0.432	
TNPEE vs. TAMA	6.825 [5.714, 7.936]		0.567	12.04	<0.001 ***		7.069 [5.856, 8.282]		0.619	11.42	<0.001 ***	
TAMA+TNPEE vs. TAMA	10.370 [9.257, 11.483]		0.568	18.25	<0.001 ***		9.575 [8.358, 10.792]		0.621	15.43	<0.001 ***	
Age × TNPEE	0.265 [−0.256, 0.786]		0.266	1.00	0.323		0.031 [−0.537, 0.599]		0.290	0.11	0.916	
Age × TAMA+TNPEE	0.345 [−0.182, 0.872]		0.269	1.29	0.203		−0.206 [−0.780, 0.368]		0.293	−0.70	0.486	
Model fit	R^2^ = 0.833			F(5,71) = 71.06			R^2^ = 0.784			F(5,71) = 51.62		
Interaction block	ΔF(2,71) = 0.943			*p* = 0.394, η^2^p = 0.026			ΔF(2,71) = 0.350			*p* = 0.706, η^2^p = 0.010		

Multiple linear regression including age (mean-centered at 8.02 years), treatment group (TAMA = reference), and Age × Treatment interaction terms. B = unstandardized regression coefficient; SE = standard error; t = t-statistic; *p* = two-tailed. The interaction block row reports the partial F-test (ΔF) and partial eta-squared (η^2^p) for the joint contribution of the two interaction terms. No significant Age × Treatment interaction was found for any subscale (all *p* > 0.18). 95% confidence intervals for regression coefficients are reported in brackets. Significance codes: *** *p* < 0.001, ** *p* < 0.01, * *p* < 0.05. Abbreviations: TAMA = Aquatic Motor Activities Therapy; TNPEE = Neuropsychomotor Therapy of Early Development; VABS-II = Vineland Adaptive Behavior Scales, Second Edition.

**Table 6:** Simple slopes: regression of chronological age on VABS-II change scores (ΔT0 → T3), estimated separately within each treatment group.

VABS-II Subscale	TAMA (n = 26)				TNPEE (n = 26)				TAMA+TNPEE (n = 25)			
	B	SE	t	*p*	B	SE	t	*p*	B	SE	t	*p*
VABS-II Composite	0.231 [0.068, 0.394]	0.083	2.80	0.010 **	0.133 [−0.045, 0.311]	0.091	1.46	0.157	0.135 [−0.065, 0.335]	0.102	1.33	0.198
VABS-II Communication	0.445 [0.147, 0.743]	0.152	2.93	0.007 **	0.021 [−0.355, 0.397]	0.192	0.11	0.912	0.131 [−0.222, 0.484]	0.180	0.73	0.474
VABS-II Socialization	0.378 [0.047, 0.709]	0.169	2.23	0.035 *	0.114 [−0.276, 0.504]	0.199	0.57	0.574	0.170 [−0.298, 0.638]	0.239	0.71	0.485
VABS-II Daily Living	−0.049 [−0.396, 0.298]	0.177	−0.28	0.784	0.216 [−0.170, 0.602]	0.197	1.09	0.285	0.296 [−0.102, 0.694]	0.203	1.46	0.159
VABS-II Motor	0.152 [−0.250, 0.554]	0.205	0.74	0.465	0.183 [−0.172, 0.538]	0.181	1.01	0.323	−0.053 [−0.523, 0.417]	0.240	−0.22	0.826

Simple OLS regression within each treatment group. b = unstandardized slope (VABS-II points per year of age); SE = standard error. Significant slopes indicate age-dependent adaptive gains within that group. 95% confidence intervals for regression coefficients are reported in brackets. Significance codes: ** *p* < 0.01, * *p* < 0.05. Abbreviations: TAMA = Aquatic Motor Activities Therapy; TNPEE = Neuropsychomotor Therapy of Early Development; VABS-II = Vineland Adaptive Behavior Scales, Second Edition.

**Table 7:** ANCOVA main-effects model: partial eta-squared effect sizes for age and treatment group on VABS-II change scores (ΔT0 → T3).

VABS-II Subscale	Age Effect		TNPEE (n = 26)		Model R^2^
	F(1,73)	*p*/η^2^p	F(2,73)	*p*/η^2^p	
VABS-II Composite	10.865	0.002/0.130 [0.02, 0.28]	764.47	<0.001/0.954 [0.93, 0.97]	0.955
VABS-II Communication	4.760	0.032/0.061 [0.00, 0.19]	182.69	<0.001/0.833 [0.76, 0.87]	0.834
VABS-II Socialization	4.130	0.046/0.054 [0.00, 0.18]	188.77	<0.001/0.838 [0.75, 0.87]	0.839
VABS-II Daily Living	1.507	0.224/0.020 [0.00, 0.12]	176.71	<0.001/0.829 [0.75, 0.87]	0.829
VABS-II Motor	0.705	0.404/0.010 [0.00, 0.10]	131.00	<0.001/0.782 [0.69, 0.83]	0.782

Main-effects ANCOVA (no interaction terms). F-statistics and partial eta-squared (η^2^p) reported for each predictor. 95% confidence intervals for η^2^p (non-central F distribution method) are reported in brackets. Treatment group accounts for the vast majority of variance (η^2^p = 0.78–0.95). Age shows a small but significant main effect for Composite, Communication, and Socialization, driven primarily by the TAMA group. Abbreviations: TAMA = Aquatic Motor Activities Therapy; TNPEE = Neuropsychomotor Therapy of Early Development; VABS-II = Vineland Adaptive Behavior Scales, Second Edition.

**Table 8:** ASD severity level transitions from baseline (T0) to 18 months (T3) by treatment group and age stratum.

Group/Age Stratum	n	ASD Level T0 (1/2/3)	ASD Level T3 (1/2/3)	Mean Δ Level	*p*-Value
TAMA—≤6 years	8	0/4/4	4/4/0	1.00 ± 0.00	0.113
TAMA—>6 years	18	5/8/5	13/5/0	0.72 ± 0.46	
TAMA+TNPEE—≤6 years	3	1/1/1	2/1/0	0.67 ± 0.58	0.430
TAMA+TNPEE—>6 years	22	3/8/11	11/11/0	0.86 ± 0.35	
TNPEE—≤6 years	5	1/2/2	3/2/0	0.80 ± 0.45	0.557
TNPEE—>6 years	21	2/14/5	16/5/0	0.90 ± 0.30	

Mean Δ Level = mean reduction in ASD severity level (positive = improvement). Between-age-stratum differences: Mann–Whitney U (two-tailed). Level distribution reported as counts (Level 1/Level 2/Level 3). Formal tests of the overall T0 → T3 shift within each treatment group (collapsed across age strata): McNemar-Bowker test of marginal homogeneity on the T0 → T3 transition table—TAMA χ^2^(2) = 21.0, *p* < 0.0001; TAMA+TNPEE χ^2^(2) = 21.0, *p* < 0.0001; TNPEE χ^2^(2) = 23.0, *p* < 0.0001; Friedman test across all four repeated time points (T0–T3)—TAMA χ^2^(3) = 36.9, *p* < 0.0001; TAMA+TNPEE χ^2^(3) = 38.7, *p* < 0.0001; TNPEE χ^2^(3) = 42.3, *p* < 0.0001. Abbreviations: ASD = Autism Spectrum Disorder; TAMA = Aquatic Motor Activities Therapy; TNPEE = Neuropsychomotor Therapy of Early Development.

## Data Availability

The data presented in this study are available on reasonable request from the corresponding author. Data are not publicly available due to privacy and ethical restrictions related to pediatric clinical data.
